# Ranolazine: safe and effective in a patient with hypertensive cardiomyopathy and multiple episodes of electrical storm

**DOI:** 10.1002/ccr3.1019

**Published:** 2017-06-02

**Authors:** Panagiotis Margos, Nikolaos Margos, Nadiya Mokadem, Ilias Patsiotis, Athanasios Kranidis

**Affiliations:** ^1^ ^st^1^st^ Cardiology DepartmentGeneral Hospital of Nikea‐Piraeus “Agios Panteleimon”Nikea‐PiraeusGreece

**Keywords:** Antiarrhythmic, defibrillator, electrical storm, ranolazine

## Abstract

Among implantable cardioverter‐defibrillator (ICD) recipients, there are patients with recurrent episodes of electrical storm (ES), retractable to the optimal antiarrhythmic drug therapy or invasive ablation procedures. A relatively novel anti‐ischemic drug with also antiarrhythmic properties, ranolazine, may effectively suppress ventricular arrhythmias in such patients for a long period of time.

## Introduction

Electrical storm (ES) is an unstable and potentially lethal medical condition of heart rhythm, characterized by at least three episodes of sustained ventricular tachycardia (VT) or ventricular fibrillation (VF) in a period of 24 h, requiring acute medical intervention. Typically, ES may occur during the acute phase of ST elevation myocardial infarction. In the modern era of implantable cardioverter defibrillators (ICDs), ES is even more common (5–20% of ICD recipients 1, 2), especially among patients treated with ICDs for secondary prevention after a first episode of sustained ventricular arrhythmia (VA).

Regarding drug therapy for secondary prevention of VA, b‐blockers in combination with amiodarone is the cornerstone in patients with at least moderate structural heart disease 3, 4. Although short‐term effectiveness of drug therapy is generally high, especially with the addition of mexiletine 5 in resistant cases, toxicity with multiple side effects of both amiodarone and mexiletine attenuates the favorable outcomes in the long term. Radiofrequency (RF) ablation is routinely applied in patients with ICD and drug‐refractory VA, but its long‐term efficacy is also not too high 6, 7. Thus, a small subgroup of ICD recipients develops drug‐ and ablation‐refractory VA. These patients with inevitably high burden of ICD shocks present with impaired quality of life and increased mortality, due to the deleterious effect of multiple shocks in psychic sphere and myocardium, respectively. Further therapeutic approach in such patients is challenging. Literature presents limited data and suggestions, including off‐label drug administration.

## Case Report

A 75‐year‐old man with NYHA‐II class systolic heart failure due to hypertensive cardiomyopathy (history of long‐lasting arterial hypertension, concentric left ventricular hypertrophy with diffuse hypokinesia – LVEF = 40% in echocardiographic study and extreme coronary artery tortuosity without significant atheromatic disease in coronary angiography) underwent an ICD implantation in March 2013, after an episode of sustained VT with hemodynamic collapse, which required urgent cardioversion.

**Figure 1:**
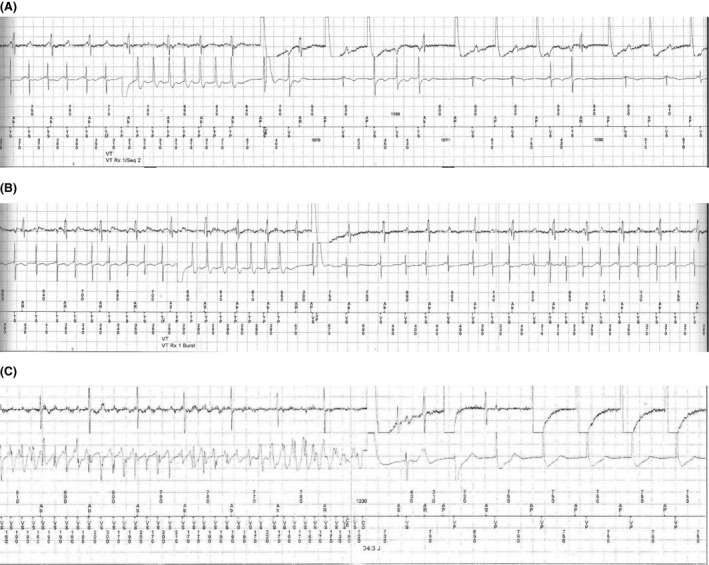
(A) Episode of sustained ventricular tachycardia (cycle length 380 ms) which terminates after application of atnitachycardia pacing. (B) Episode of sustained ventricular tachycardia (cycle length 350 ms), resistant to atnitachycardia pacing. (C) Episode of ventricular fibrillation which terminates after application of high‐energy DC shock.

Soon after the implantation, ES occurred with multiple episodes of sustained VT, requiring either antitachycardia pacing (ATP) or shock therapy for sinus rhythm restoration (Fig. 1). Intravenous amiodarone was administered acutely, followed by chronic per os intake. New episodes of ES occurred during the following period, with no detectable triggering condition. Two transvenous ablation procedures were performed within 6 months, both without success. In both electrophysiological studies, ventricular stimulation was characterized by the induction of at least five different morphologies of sustained VT. During follow‐up, three different high‐dose b‐blocker agents were administered consecutively (carvedilol, metoprolol, or bisoprolol in combination with amiodarone), as well as sotalol, with no clinical response. Mexiletine was added for a period of only 2 months. It was prematurely discontinued due to severe central nervous system toxicity.

**Figure 2:**
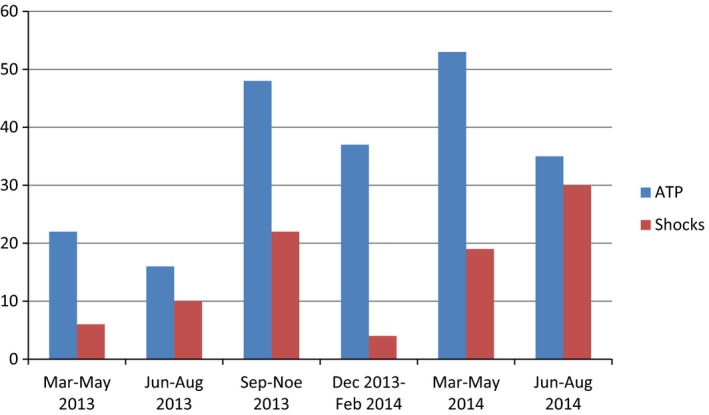
First device lifetime episodes (211 ATP, 91 shocks in total), in a period of 18 months.

In summary, during the period of 16 months after implantation, 211 appropriate ATP and 91 appropriate shocks occurred (Fig. 2), despite our attempts to reduce shock burden, through adequate adjustment of therapy‐zone parameters. More specifically, the lower limit of VT zone was gradually elevated from 136 beats per minute (bpm) t*ο* 162 bpm (detection: 20 beats, redetection: 12 beats), as even long‐lasting slow VT episodes were well tolerated, with no obvious hemodynamic deterioration. Additionally, the application of burst sequences (three sequences, 88%, 10 pulses) of VT zone was proven to be (more or less) ineffective. Thus, burst sequences were replaced by a more aggressive (and more effective) protocol of 3 plus 3 Ramp+ sequences (84%/78%/75%, eight pulses and 75%/69%/66%, eight pulses, respectively). In general, ATP and shocks were distributed normally in time, without long periods of recession. As a consequence, energy depletion of the device occurred prematurely (September 2014, device replacement). The heart rate spectrum of VTs was wide (120–230 bpm) and many episodes of sustained VT were only monitored without any intervention, displaying R‐R interval above the therapy zones (heart rate 120–162 bpm, monitor‐only zone). During this period, the patient underwent multiple hospitalizations, mainly receiving I.V. amiodarone. His quality of life clearly deteriorated, with new‐onset symptoms of anxiety and depression (psychiatric consultation was also performed). Despite multiple shock therapies, four consecutive echocardiographic studies were fortunately comparable to the initial one, with no clear deterioration of left ventricular systolic function.

**Figure 3:**
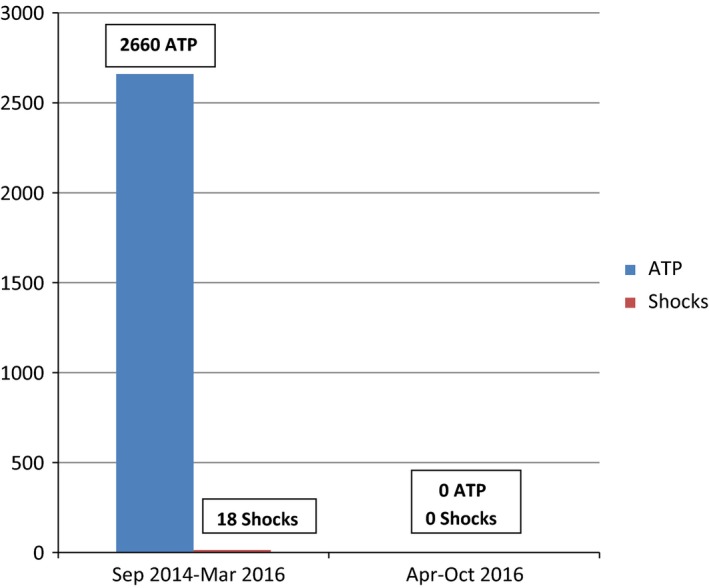
Second device episodes for a total period of 26 months. Extremely high burden of ATP (2660) and only 18 shocks, during the initial period of 19 months (Sep 2014 to Mar 2016). No ATP or shock occurred during the following period of 7 months (Apr‐Oct 2016, patient under ranolazine).

After device replacement, new ES episodes occurred, as expected. The pattern of VTs and subsequent device interventions was somewhat different, with lower mean heart rate of VT episodes, many hundreds of ATP and only few shocks, always appropriate (Fig. 3). Fifteen months after device replacement, subclinical hyperthyroidism was detected (December 2015, no previous history). Amiodarone was replaced by flecainide, with no clear clinical response (new episodes of VT occurred, with even lower mean heart rate, new therapeutic interventions – mainly ATP – were detected).

Three months after amiodarone discontinuation, in March 2016, ranolazine was added to the antiarrhythmic therapy (patient under carvedilol 12.5 mg × 3 and flecainide 100 mg × 2), with up‐titration to the dose of 750 mg × 2. Surprisingly, during the following 7 months (March to October 2016), no VT or VF episode occurred, in complete contrast to the displayed history of the previous 3 years, since first ICD implantation, with a total burden of 2871 ATP and 109 shocks, all appropriate (Fig. 3). It should be mentioned that no reversible underlying arrhythmogenic conditions (other than cardiomyopathy) were ever detected in our patient. Additionally, the patient's adherence to medical advisory was reliable and constant during the long period of 3.5 years of follow‐up. Currently, the patient is under a good psychologic and a stable functional status (NYHA‐II).

## Discussion

Electrical storm is an increasingly common, life‐threatening clinical situation, mainly presented in ICD recipients. Attending physicians should always be concentrated to the aim of primary or secondary prevention of ES during close follow‐up. This goal is served not only through drug suppression of arrhythmias, but also with the parallel attempt for detection and restoration of any potentially reversible underlying condition that contributes to arrhythmogenesis (i.e., reversible ischemia, electrolytic disorders, inadequate or inconsistent drug intake in total, dietary incompliance).

Unfortunately, few antiarrhythmic drugs are currently available for the prevention or suppression of potentially lethal VA in patients with cardiomyopathy. Beyond b‐blockers and amiodarone, only sotalol can be used in patient with mildly reduced LVEF, like our patient 8. For the rest of the patients with at least moderate left ventricular systolic dysfunction, administration of other antiarrhythmic agents not only lacks favorable clinical outcome, but even displays deleterious (proarrhythmic) effect 9. Nevertheless, flecainide (like our case) and quinidine may contribute to the reduction in VA burden in selected patients with structural heart disease, under the protection of ICD 8, 9, 10.

Ranolazine is a relatively novel drug that intervenes in transmembrane cardiac action potential by ion current inhibition. The resultant reduction in intracellular Na^+^ concentration inhibits partially the Na^+^/Ca^++^ exchange current, preventing the deleterious effect of intracellular Ca^++ ^overload under the trigger of ischemia. This indirect decrease in intracellular Ca^++^ concentration is responsible for the well‐documented antianginal effect of ranolazine 11, 12. In the field of clinical studies, ranolazine is presented as an effective, well‐tolerated, and safe drug, in patients with coronary artery disease and residual reversible ischemia 13, 14.

Beyond anti‐ischemic properties, ranolazine displays remarkable similarity with class I and class III antiarrhythmic drugs, as a pure ion current inhibitor 15, 16. Experimental evidence emphasizes the increase in VF threshold and the suppression of ischemia‐induced arrhythmias by ranolazine 17, 18. According to MERLIN‐TIMI 36 trial, ranolazine suppresses VA during the first week after admission for non‐ST elevation acute coronary syndrome 14. In such patients, even short VT episodes of only few beats are associated with the risk of sudden cardiac death 19.

Implantable cardioverter defibrillator recipients who present with antiarrhythmic drug‐refractory VA and recurrent ICD shocks provide an urgent therapeutic challenge. Limited therapeutic options are available, as mentioned before. In such intractable cases, previous reports support the adjunct role of ranolazine to the usual medical care. Bunch et al. 20 reported the effectiveness of ranolazine in 11 of 12 patients with refractory VT. Notably, 10 of them had ischemic heart disease. Ranolazine‐induced VA suppression has also been reported in patients with nonischemic cardiomyopathy 10, 20. Additionally, in patient with quite frequent premature ventricular complexes (PVC > 10%), ranolazine decreased PVC burden, approximately 60%, especially among individuals with impaired left ventricular function 21.

Atrial fibrillation (AF) is another potential therapeutic target for ranolazine. A trend for reduced episodes of new‐onset AF was already mentioned in MERLIN‐TIMI 36 trial 14. Several subsequent reports highlight the positive effect of ranolazine against atrial fibrillation in specific populations: post‐CABG, for the conversion of recent‐onset atrial fibrillation or in cardioversion‐resistant patients 15, 22, 23, 24, 25, 26, 27, 28. Moreover, RAFFAELLO trial 29 demonstrated that ranolazine in doses of 500 and 750 mg reduced AF recurrences compared to placebo (borderline significance).

In patients with long QT syndrome, ranolazine shortens the prolonged QTc and suppresses early afterdepolarizations and TdP episodes 30, 31, 32. Currently, ranolazine displays a IIb indication (as add‐on therapy to b‐blocker) in LQTS3 patients with a QTc > 500 msec, in order to shorten the QT interval 33.

A recent review article summarizes the antiarrhythmic role of ranolazine in general, emphasizing on its relatively few side effects, in comparison with other pure antiarrhythmic drugs 34. Currently, a randomized trial (Ranolazine Implantable Cardioverter Defibrillator – RAID trial), investigates the efficacy of routine ranolazine administration, beyond standard antiarrhythmic therapy, in patients with ICDs 35.

Regarding our patient, the total burden of VA and ES was extremely high during three‐year follow‐up after the initial ICD implantation, with no long periods of recession. Amiodarone toxicity (subclinical hyperthyroidism), in combination with ablation failure, set a deadlock in our therapeutic approach. Although flecainide is contraindicated in patients with structural heart disease (increased mortality), it is well known that it decreases the burden of VA 36. ICD presence allowed flecainide administration, which offered an initial benefit in VA suppression. Subsequently, ranolazine was added, despite the absence of significant coronary artery disease and ischemia‐triggered VA, as recent reports highlight ranolazine‐induced VA suppression, even in nonischemic cardiomyopathy 10. This is attributed to its pure antiarrhythmic effect, in the absence of active ischemia. The effectiveness of ranolazine was impressive (VA elimination for an ongoing period of 7 months), better than expected, with no notable side effects. Notably, left ventricular systolic function and functional class (NYHA‐II) were not affected during the three‐year unstable period, while psychiatric consultation contributed to the restriction of anxiety and depression symptoms.

In conclusion, this is a quite rare case of an ICD recipient with an extremely high burden of VA and multiple episodes of ES, despite the appropriate antiarrhythmic therapy. After amiodarone and mexiletine discontinuation, due to toxicity, ranolazine administration, in combination with carvedilol and flecainide, offered total suppression of VA, for an ongoing period of 7 months. Ranolazine may be a quite valuable therapeutic option in such patients with multiple, retractable ventricular arrhythmias and episodes of electrical storm.

## Authorship

PM, NM, NM, IP, and AK: were all contributors to the study design/conception and data acquisition, analysis, and interpretation.

## Conflict of Interest

None declared.
